# Spontaneous electromagnetic induction promotes the formation of economical neuronal network structure via self-organization process

**DOI:** 10.1038/s41598-019-46104-z

**Published:** 2019-07-04

**Authors:** Rong Wang, Yongchen Fan, Ying Wu

**Affiliations:** 1 College of Science, Xi’an University of Science and Technology, Xi’an, 710054 China; 2 State Key Laboratory for Strength and Vibration of Mechanical Structures, Shaanxi Engineering Laboratory for Vibration Control of Aerospace Structures, School of Aerospace, Xi’an Jiaotong University, Xi’an, 710049 China

**Keywords:** Dynamical systems, Network models, Biological physics, Complex networks

## Abstract

Developed through evolution, brain neural system self-organizes into an economical and dynamic network structure with the modulation of repetitive neuronal firing activities through synaptic plasticity. These highly variable electric activities inevitably produce a spontaneous magnetic field, which also significantly modulates the dynamic neuronal behaviors in the brain. However, how this spontaneous electromagnetic induction affects the self-organization process and what is its role in the formation of an economical neuronal network still have not been reported. Here, we investigate the effects of spontaneous electromagnetic induction on the self-organization process and the topological properties of the self-organized neuronal network. We first find that spontaneous electromagnetic induction slows down the self-organization process of the neuronal network by decreasing the neuronal excitability. In addition, spontaneous electromagnetic induction can result in a more homogeneous directed-weighted network structure with lower causal relationship and less modularity which supports weaker neuronal synchronization. Furthermore, we show that spontaneous electromagnetic induction can reconfigure synaptic connections to optimize the economical connectivity pattern of self-organized neuronal networks, endowing it with enhanced local and global efficiency from the perspective of graph theory. Our results reveal the critical role of spontaneous electromagnetic induction in the formation of an economical self-organized neuronal network and are also helpful for understanding the evolution of the brain neural system.

## Introduction

Neurons in the nervous system embedded in neural networks are connected via synapses where the efficiency of the network’s connectivity pattern is crucial for effective communication of neural information^1,2^. Developed through evolution, these neuronal networks are optimized to economically transfer as much neural information as possible with a low energy cost^1–6^; such a network can be well described by a small-world network model with a high efficiency of information communication on both local and global scales^2,7,8^. Due to the synaptic plasticity, macroscopic brain functions, such as learning, memory, and similar cognitive processes, promote the self-organization of microscopic neuronal networks to dynamically match the functional demands^9–14^. Such a self-organized structure possessing both small-world and scale-free properties has been found to be a more realistic way to characterize the real neuronal network^15^ and to have a considerable effect on the collective dynamics of neurons^16–22^. For example, a microscopic self-organized neuronal network has higher coherence resonance, stochastic resonance and efficiency of information transmission than a globally coupled network or a random network while they have the same mean synaptic weight^16^. Therefore, exploring how the neural system self-organizes to optimize its network structure to possess economical topological properties is helpful for studying collective neuronal dynamics and is also important for a more realistic understanding of the brain evolution.

The self-organized structure is originated from the feedback coupling between global dynamics and local neuronal networks through the biologically seen spike-timing-dependent plasticity (STDP) rule, which has been observed in various vivo and vitro experiments^23–30^. This STDP rule updates synaptic weights between neurons according to the relative timing between pre- and postsynaptic action potentials on a millisecond timescale^29^. If the firing time for the presynaptic neuron is ahead of that for the postsynaptic neuron, the synaptic connection is enhanced; otherwise, it is weakened^23^. The modulated synapses in turn affect the neural responses. This feedback coupling between global network dynamics and local neuronal circuits is an important operating mode of the brain^31^ and is closely related to the mechanisms of learning and memory^32,33^. However, the neuronal electric activities generated by the transmembrane flow of ions not only modulate the synaptic connection but also inevitably produce a time-varying electric field as well as a magnetic field, according to Maxwell’s theory of electromagnetic induction^34–36^. This spontaneous magnetic field around neurons may be the foundation of brain transferring sensory stimulus via complex electromagnetic flows to the cortex^37^ and has significant effects on the dynamical properties of neurons and neuronal networks^34,38–41^; for example, it induces multiple firing modes of neurons^42,43^, promotes the double coherence resonance, inhibits the stochastic resonance^40^ and modulates spatiotemporal patterns^44^. Meanwhile, magnetic field interactions between neurons support a potential spatial channel for neural information transmission^45,46^ and can significantly modulate signal communications between neurons^45,47^, induce firing synchronization^48,49^, trigger complex mode transitions of electrical activities^50–53^, and even offset the effect of a blocked potassium ion channel on the collective dynamics^54^. Furthermore, this spontaneous electromagnetic induction can induce abundant chaotic dynamics in a previously steady-state neural network; this phenomenon has been experimentally proven with an equivalent electric circuit model of the neural network in PSpice^53^. More importantly, spontaneous electromagnetic induction in neurons provides a reasonable and effective path to study the effect of external electromagnetic fields on neural system functions^48,55–58^. For example, the electromagnetic radiation can induce diverse synchronizations^55,59^, stochastic resonance^60^, rhythm disorder^59^ and mode transitions of electrical activities^56^ in the neuronal network; in particular, an optimal electromagnetic radiation intensity can induce the occurrence of stochastic resonance and a maximal neuronal response to weak signals^55^. Even the spontaneous electromagnetic induction (i.e., negative magnetic feedback on neurons and magnetic coupling between them) has great effects on the dynamics of neurons and neuronal networks, its complex modulation on the self-organization of the neuronal network is still lacking.

Here, we investigate the effects of spontaneous electromagnetic induction on the evolutionary process and topological properties of the self-organized neuronal network at the microscopic scale. We first study how the negative feedback of magnetic fields on neurons and the magnetic coupling between them affect the self-organization process in the neuronal network, and then we utilize graph theory to explore the topological properties of the network. We find that spontaneous electromagnetic induction has complex effects on the evolutionary process and topological structure of the self-organized neuronal network. In particular, negative the feedback from a magnetic field can induce the economical neuronal network structure by reconfiguring synaptic connections during the self-organization process, and the magnetic coupling can further promote the formation of economical self-organized network structures.

## Results

In this paper, the Euler-Maruyama algorithm is used to solve the differential equations with a time step of 0.005 ms and a total time of 200 ms. The 80 excitatory neurons and 20 inhibitory neurons considered in the network are initially globally coupled by chemical synapses, where the synaptic weight is set to *g*_*max*_/2 for excitatory synapses and to 3 *g*_*max*_/2 for inhibitory synapses^16–18^. During the self-organization process, the weights of excitatory synapses are updated based on the STDP rule, but the weight remains constant for inhibitory synapses^16–18^.

### Negative feedback of spontaneous magnetic field inhibits the self-organization process

**Figure 1:**
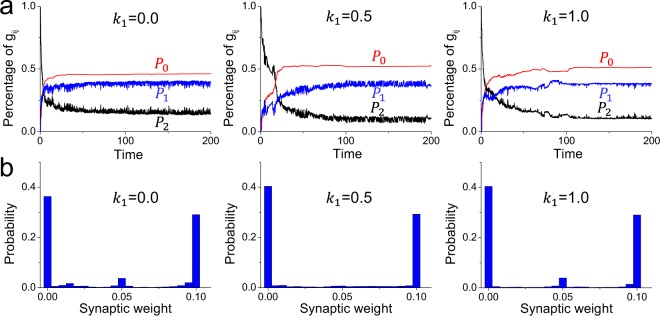
(**a**) The evolutionary processes of *P*_0_, *P*_1_ and *P*_2_ under negative feedback of different strengths. Note that for the simulation time longer than 150 ms, *P*_0_, *P*_1_ and *P*_2_ keep largely stable, as does the neuronal network structure. (**b**) The probability distribution of synaptic weights in dynamically self-organized neuronal networks. Here, the dynamic network structures are obtained from 150 ms to 200 ms with a sampling step of 0.05 ms; these structures are also used for subsequent calculations without explicit explanation.

To investigate the self-organization process of the neuronal network structure as modulated by spontaneous electromagnetic induction, we first focus on the negative feedback of magnetic fields on neurons rather than the magnetic coupling between them, i.e., fixing the magnetic coupling strength *D* = 0 and varying the negative feedback strength *k*_1_. Meanwhile, to simply investigate the evolutionary process of the self-organized neuronal network, we use *P*_0_ to denote the percentage of synapses with weights in the range [0, 0.1 *g*_*max*_] (weak coupling), *P*_1_ to represent the percentage of synapses with weights in the range [0.9 *g*_*max*_, *g*_*max*_] (strong coupling), and *P*_2_ to stand for all other cases^16,18^. Due to the competition between heterogeneous neurons, synaptic connections from high-active neurons to low-active neurons are enhanced, but they are weakened from low-active neurons to high-active ones^16,17^. Thus, *P*_0_ and *P*_1_ increase during the self-organization process, whereas *P*_2_ decreases^16,18^ (Fig. 1a), reflecting the modulation of chemical synapses by neuronal electric activities and the formation of a sparse self-organized structure. As *P*_0_, *P*_1_ and *P*_2_ gradually reach stable values, the neuronal network achieves a dynamically stable state (i.e., longer than 150 ms; see Fig. 1a) and self-organizes into a directed-weighted network structure.

**Figure 2:**
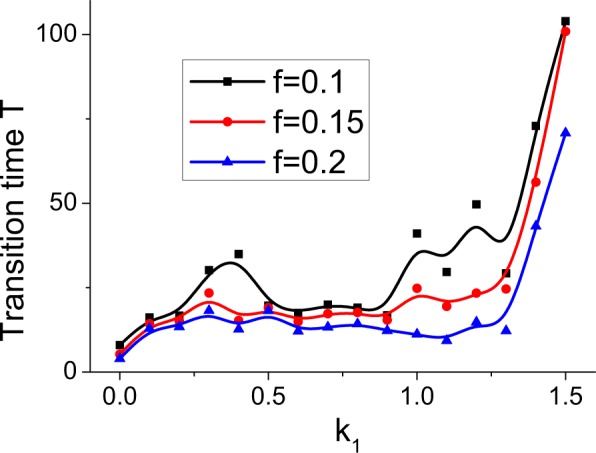
The transition time *T* versus the negative feedback strength *k*_1_ for different artificial fluctuation coefficients *f*, which reflects the relatively evolutionary speed of self-organization process.

Despite the similar evolutionary processes of neuronal networks for different negative feedbacks, the spontaneous magnetic field significantly affects the self-organization speed. We use *P*_1_ to measure the evolutionary speed of the neuronal network due to its relatively small fluctuation in the dynamically stable state (see Fig. 1a). Assuming that $\overline{{P}_{1}}$ is the mean value of *P*_1_ in the dynamically stable state from 150 ms to 200 ms and *f* is an artificial fluctuation coefficient of *P*_1_, if *P*_1_ always fluctuates within a range of $[(1-f)\overline{{P}_{1}},(1+f)\overline{{P}_{1}}]$ over a transition time *T*, we claim that the neuronal network has achieved a dynamically stable state on the time scale *T*. Thus, the neuronal network has a higher self-organization speed for a shorter time *T*. This measure is apparently dependent on the fluctuation coefficient *f* and reflects the relative evolutionary speed. To present more reliable results, we calculate the transition times *T* for different fluctuation coefficients (Fig. 2). As *k*_1_ increases, the transition time *T* tends to increases — in particular, neuronal networks with *k*_1_ > 0.0 have significantly higher transition times than that for *k*_1_ = 0.0 — and this tendency is robust for different fluctuation coefficients (Fig. 2). These results indicate that spontaneous coupling between the magnetic field and the membrane potential slows down the self-organization process of the neuronal network.

### Negative feedback of spontaneous magnetic field induces a more homogeneous directed network structure

**Figure 3:**
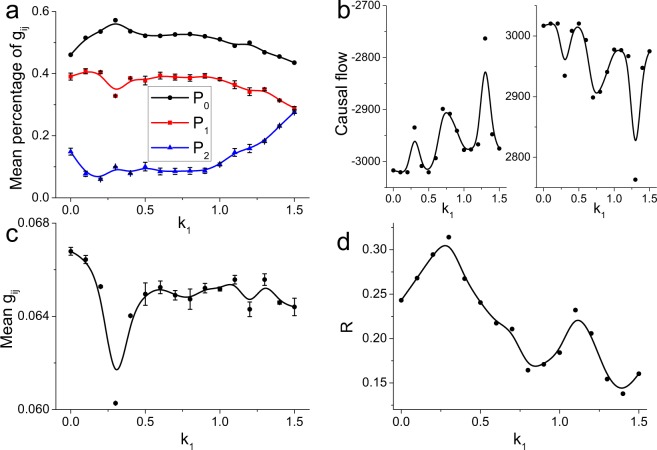
(**a**) The mean values of *P*_0_, *P*_1_ and *P*_2_ in dynamic neuronal networks for different values of *k*_1_. (**b**) The causal flows and (**c**) the mean values of synaptic weights of dynamically self-organized neuronal networks under negative feedback of different strengths. Here, the positive causal flows are averaged from the causal source neurons and the negative ones are averaged from the causal sinks. These results are first calculated for each network individually from 150 ms to 200 ms with a sampling step of 0.05 ms and then averaged. (**d**) The factor of synchronization (defined in Eq. 12) in dynamic neuronal networks, which is calculated from 150 ms to 200 ms and reflects the stronger network synchronization for a higher value.

By affecting the self-organization process, spontaneous magnetic field also has an influence on the synaptic weights in the stable self-organized neuronal network. To analyze the synaptic weight, we calculate the mean values of *P*_0_, *P*_1_ and *P*_2_ in the stable state from 150 ms to 200 ms with a sampling step of 0.05 ms. As *k*_1_ increases, the mean *P*_0_ first increases and then decreases, the mean *P*_2_ shows a completely opposite variational trend, and the mean *P*_1_ nearly always decreases (Fig. 3a), reflecting nonmonotonic and complex modulation on synapses. For moderate negative feedback (e.g., *k*_1_ = 0.5), neuronal networks have smaller *P*_2_ and larger *P*_0_ values than those for *k*_1_ = 0.0, reflecting that the negative feedback from the magnetic field promotes the modulation of synaptic connections, such that synapses with intermediate weights (i.e., *g*_*ij*_ = 0.05) are reconfigured into weak synapses (see Fig. 1b). For strong negative feedback (e.g., *k*_1_ = 1.0), neuronal networks have smaller *P*_0_ and *P*_1_ values but larger *P*_2_ values than those for *k*_1_ = 0.0 (Fig. 3a), indicating that in this case, the magnetic field instead weakens the modulation of synaptic connections, such that many synapses with intermediate weights are not modulated (see Fig. 1b for *k*_1_ = 1.0). Thus, spontaneous magnetic field first promotes the modulation of synaptic connections in the stable self-organized neuronal network and then inhibits the modulation.

During the STDP updating process, directed synaptic connections are modulated by the firing lag between the pre- and postsynaptic neurons, which actually reflects a kind of causal relationship between them. Spontaneous magnetic field has a great influence on the modulation of synaptic connections and will consequently affect the causal relationships between neurons. We utilize a causal flow (defined in Eq. 8) to measure the causal relationship among neurons in self-organized neuronal networks. A neuron with a higher positive causal flow has a greater causal influence on others and is more likely to be a “causal source” in the neuronal network. By contrast, a neuron with a small negative causal flow is greatly affected by other neurons and is called a “causal sink” of the network^61^. Previous studies have found that a neuron with higher excitability tends to have a larger out-degree and a smaller in-degree^16,17^, i.e., is more likely to be a causal source, and a neuron with lower excitability is more likely to be a causal sink. And the local excitation can promote the synchronous propagate of neural activities through the heterogeneous self-organized neuronal network^62^. Due to the negative feedback exerted by magnetic fields on firing activities, it is reasonable to suspect that spontaneous magnetic field will enhance the synaptic inputs to high-active neurons and weaken their outputs. We find that as the negative feedback becomes stronger, the negative causal flow increases and the positive causal flow decreases (Fig. 3b), indicating that the negative feedback from a magnetic field weakens the causal relationship by decreasing the neuronal excitability. These results also reveal that spontaneous magnetic field induces a more homogeneous directed self-organized network structure with less heterogeneity between in- and out-synaptic weights of neurons.

Despite the spontaneous magnetic field inhibits the synchronization of the neuronal network with undirected and constant synapses^49^, it has quite complex effects on the synchronization of the directed self-organized network by modulating the feedback between global network dynamics and local neuronal circuits. As the negative feedback strength increases, the mean synaptic weight in the self-organized neuronal network first decreases to the minimum value (i.e. *k*_1_ = 0.3, Fig. 3c), accompanied by a increase of network synchronization (i.e. a higher factor of synchronization, see Fig. 3d). In fact, in the directed and heterogeneous self-organized network, synaptic connections from high-active neurons to low-active neurons are significantly larger than those from low-active neurons to high-active neurons^16,17^. While the weak connections are very small, low-active neurons are mainly driven by high-active neurons and thus the network can achieve high synchronization. At *k*_1_ = 0.3, spontaneous electromagnetic field further increases the percentage of weak synaptic connections from low-active neurons (i.e. higher *P*_0_, Fig. 3a) and consequently enhances the firing synchronization (Fig. 3d). With the negative feedback strength increasing, the percentage of weak synapses are decreased (i.e. lower *P*_0_, Fig. 3a) and the mean synaptic weight increases (Fig. 3c), accompanied with more homogeneity between in- and out-synaptic weights of neurons (Fig. 3b). In this case, high-active neurons would receive more synaptic inputs from low-active neurons and their dominant roles are weakened. Thus, the network synchronization decreases (Fig. 3d). These results clearly reveal that due to the complex modulation on synaptic connections, spontaneous magnetic field significantly decreases the network synchronization by inducing a more homogeneous directed-weighted self-organized neuronal network.

### Negative feedback of spontaneous magnetic field induces the formation of economical network structure

**Figure 4:**
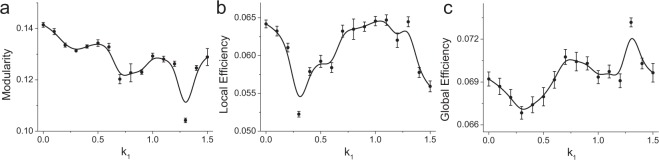
The (**a**) modularity, (**b**) local efficiency and (**c**) global efficiency of dynamically self-organized neuronal networks under negative feedback of different strengths.

We now investigate the effect of spontaneous magnetic field on the topological properties of self-organized neuronal networks from the perspective of graph theory. Modularity is the most obvious property of neuronal networks^18,63^ and has great effects on the network dynamics^64^. During the self-organization process, neurons with similar firing properties are more likely to be organized into a module through competition, such that neurons in the same module have relatively higher synaptic weights than those in different modules. This phenomenon ensures more efficient communication of neural information within modules^63,65^. The modularity (defined in Eq. 9) measures the division of a network into modules, where networks with high modularity have denser modules^18,63^. We observe that as the feedback strength increases, the modularity decreases (Fig. 4a), indicating that there are sparse modules in the self-organized neuronal network. Thus, spontaneous magnetic field can decrease the modularity of the network structure by inducing the formation of a more homogeneous directed self-organized neuronal network.

However, synaptic weights between modules should also be enough strong to support the global integration of neural information, which will inevitably wipe away the modular structure^63,65^. The most economical structure should have a moderate degree of modularity, corresponding to a relatively high efficiency of neural information flow within and between modules. We observe that as the negative feedback strength initially increases (e.g., *k*_1_ ≤ 0.3), the local efficiency (see Eq. 10) temporarily decreases (Fig. 4b), indicating that the self-organized neuronal network has less ability to transfer neural information in local circuits due to the increased percentage of weak synapses induced by the negative feedback from magnetic fields (i.e., increased *P*_0_, Fig. 3a). As the negative feedback strength further increases, the mean synaptic weight increases and the percentage of modulated synapses remains at a high value (i.e. low *P*_2_, see Fig. 3a), accompanied by an increase in local efficiency, reflecting the strengthening and relative optimization of synaptic connections such that neurons can more easily cluster together to transfer neural information. For higher negative feedback strengths, the percentage of modulated synapses decreases with increased *P*_2_ (Fig. 3a), and the local efficiency is again reduced to a small value, suggesting that less reconfigured synaptic connections limit the transmission of neural information in the local scale.

Considering the complex modulation of synaptic connections, the negative feedback from magnetic fields will first reduce the synaptic weights (especially for weak synapses) and then increase the number of synapses with less modulation as *k*_1_ increases. In a sparse self-organized network, the combination of the decreased synaptic weight and number of synapses will definitely result in fewer and weaker synaptic paths for the transmission of neural information, resulting in a temporary decrease in the transmission of neural information in the global scale (see Fig. 4c for *k*_1_ < 0.3). As the negative feedback strength increases, the percentage of modulated synapses decreases, accompanied by a larger number of synapses and higher mean synaptic weight that can provide more paths to transfer neural information. Specifically, the percentage of weak synapses decreases (i.e. decreased *P*_0_, Fig. 3a), suggesting more paths from low-active neurons to high-active neurons. Consequently, the global efficiency (see Eq. 11) begins to increase (Fig. 4c). Meanwhile, the synaptic weight begins to recover but is still smaller than that for *k*_1_ = 0.0, whereas the global efficiency is larger than that for *k*_1_ = 0.0. These results reflect that self-organized neuronal networks under spontaneous magnetic field may have higher ability to integrate neural information throughout the whole network.

**Figure 5:**
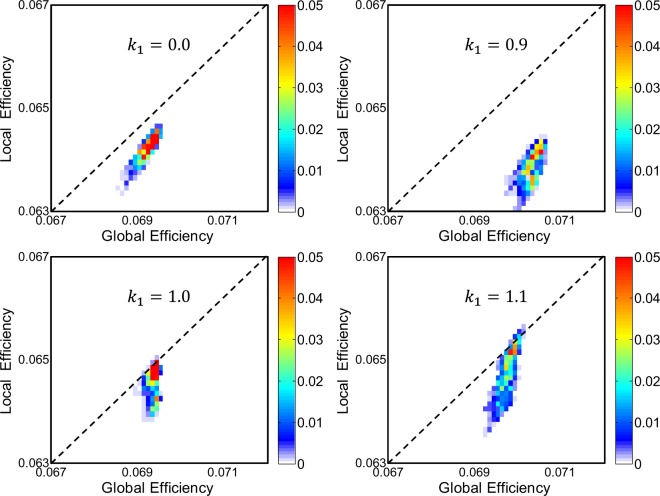
The joint probability distribution of global efficiency and local efficiency for different negative feedback strengths. The global efficiency and local efficiency are first obtained from dynamically self-organized neuronal networks, and then the joint probability distribution is plotted. The most economical network structure should have both high local efficiency and high global efficiency, i.e., the joint probability should be close to the diagonal line but tend toward the upper right corner of the panel.

In addition, it is important to notice the anomalous phenomenon that for *k*_1_ = 0.9–1.1, both the local efficiency and global efficiency are significantly larger than those for *k*_1_ = 0.0 (Fig. 4b,c), reflecting a more economical neuronal network structure from the perspective of graph theory. From the joint probability distribution of the local efficiency and global efficiency in dynamically self-organized networks, we can identify the most economical network structure, with the highest local and global efficiency (i.e., *k*_1_ = 1.1; see Fig. 5), for which the percentage of modulated synapses and the mean synaptic weight are significant smaller than those for *k*_1_ = 0.0. These results indicate that spontaneous magnetic field can optimally reconfigure synaptic connections to promote the formation of an economical neuronal network structure via the self-organization process and produce the most economical structure with a negative feedback strength of *k*_1_ = 1.1.

### Spontaneous magnetic coupling enhances the self-organization process

A spontaneously formed magnetic field not only exerts negative feedback on its corresponding neuron but also can interact with the magnetic fields of other neurons, thereby physically providing a spatial channel for the transfer of neural information^45,53^. Previous studies have found that the spatial coupling between magnetic fields has a significant influence on the collective dynamics of neurons^45,53,56^. It is thus interesting to investigate how the spatial coupling between magnetic fields modulates the self-organization process and the topological properties of the neuronal network.

**Figure 6:**
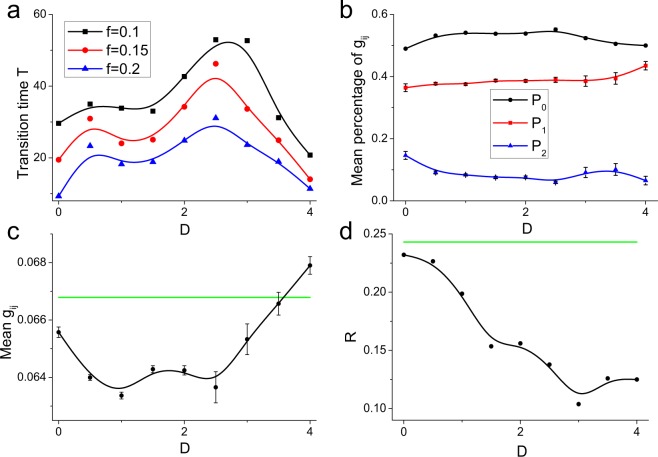
(**a**) The transition times *T* for different artificial fluctuation coefficients versus the magnetic coupling strength *D*. The (**b**) mean values of *P*_0_, *P*_1_ and *P*_2_, (**c**) mean synaptic weights and (**d**) factor of synchronization in dynamically self-organized neuronal networks from 150 ms to 200 ms with a sampling step of 0.05. The green lines represent the values of corresponding measures for the network without electromagnetic induction (i.e., *k*_1_ = 0.0).

By fixing the negative feedback strength *k*_1_ = 1.1 and varying the coupling strength *D*, we first analyze how this magnetic coupling modulates the self-organization process. As *D* increases, the transition time *T* first increases and then decreases (Fig. 6a), and this phenomenon is robust for different artificial fluctuation coefficients *f*, reflecting the complex modulation exerted by magnetic coupling on the self-organization speed. By promoting the communication of neural information among neurons^49,66^, the magnetic coupling, on the one hand, enhances the self-organization process, and on the other hand, induces higher negative feedback on neurons and thus contrarily slows down the self-organization process. These competing influences on the self-organization process result in the complex modulation of the self-organization process by the magnetic coupling (Fig. 6a). However, it is important to note that the transition time *T* under electromagnetic induction is significantly larger than that without magnetic feedback (7.995, 5.265 and 3.973 for *f* = 0.1, 0.15 and 0.2, respectively; see Fig. 2). Thus, although the magnetic coupling between neurons contributes to the self-organization process, the negative feedback exerted by magnetic fields on neurons significantly decreases the neuronal excitability and eventually inhibits the self-organization process. These results demonstrate that spontaneous electromagnetic induction slows down the self-organization process of the neuronal network.

### Spontaneous magnetic coupling contributes to a homogeneous directed self-organized neuronal network

We now investigate how the spontaneous magnetic coupling affects the synaptic connections in the directed self-organized neuronal network. As the coupling strength *D* increases, the mean *P*_0_ first increases and then decreases (Fig. 6b), which results in a temporal decrease of mean synaptic weight (Fig. 6c). However, the mean *P*_1_ always increases, which would enhance the mean synaptic weight. Indeed, the magnetic coupling between neurons contributes to the transmission of neural information^49,52,56,66,67^ and thus can promote the modulation of synaptic connections between neurons (i.e. low *P*_2_, see Fig. 6b), which decreases the mean synaptic weight. However, the enhanced negative feedback on neurons induced by magnetic coupling would also decrease the neuronal excitability, and consequently, the modulation is inhibited with increased *P*_2_ (Fig. 6b), which conversely increases the synaptic weights. This complex modulation on synapses by spontaneous magnetic coupling would result in a U-shaped curve describing the mean synaptic weight as the coupling strength *D* increases (Fig. 6c).

**Figure 7:**
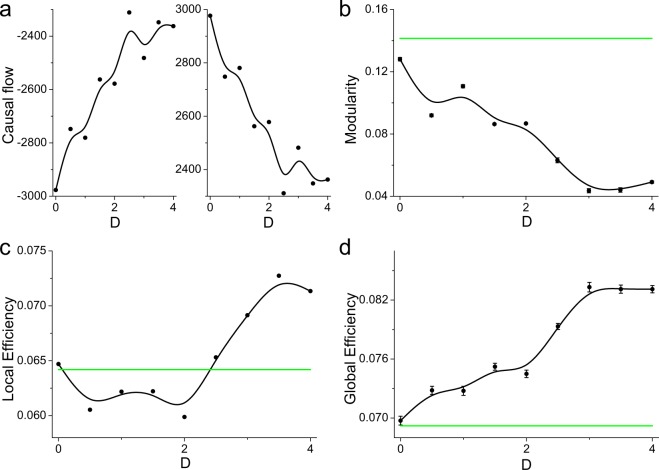
The (**a**) causal flow, (**b**) modularity, (**c**) local efficiency and (**d**) global efficiency of dynamically self-organized neuronal networks with different magnetic coupling strengths. The green lines represent the corresponding values in the neuronal network without negative magnetic feedback (i.e., *k*_1_ = 0.0).

Despite the complex modulation of synaptic weights caused by magnetic fields, we observe a continuous decrease of causal flow as the coupling strength *D* increases (Fig. 7a), indicating that the negative feedback weakens the causal relationships between neurons, consistent with the previous results in Fig. 3b. Thus, the causal relationships between neurons are mainly related to neuronal excitability (i.e., controlled by negative magnetic feedback). Meanwhile, the decreased causal flow reflects the less difference between in- and out-synaptic weights, which results in a lower network synchronization (Fig. 6d). Considering the increased percentage of strong synapses and the decreased percentage of weak synapses (Fig. 6b), it is thus reasonable to suspect that, by decreasing the neuronal excitability, spontaneous magnetic coupling may enhance the synaptic connections from low-active neurons to high-active neurons such that the number of strong synapses is improved but the network synchronization decreases. These results indicate that spontaneous electromagnetic induction results in a more homogeneous directed-weighted self-organized neuronal network structure with less causal relationship and weaker synchronization.

### Spontaneous magnetic coupling promotes the formation of economical neuronal network structure

We now investigate how the spontaneous magnetic coupling affects the topological properties of neuronal networks as formed through the self-organization process. The weakening of the causal relationship between neurons results in a less heterogeneous directed network structure in terms of the synaptic weights, which leads to decrease in network modularity (Fig. 7b). As a result, the modularity of self-organized neuronal networks further decreases for stronger magnetic coupling and is significantly smaller than that for *k*_1_ = 0.0 (Fig. 7b). Meanwhile, the number of strong synapses are increasing as the coupling strength *D* is enhanced (i.e. increased *P*_1_, Fig. 6b), which provides more strong paths to transfer neural information and results in a continuously increase in global efficiency in the directed-weighted neuronal network (Fig. 7d). Furthermore, we observe that the local efficiency first decreases and then increases as the coupling strength *D* increases (Fig. 7c), similar to the mean synaptic weight (Fig. 6c). Due to the increased percentage of weak synaptic weights induced by spontaneous magnetic coupling (i.e. increased *P*_0_, Fig. 6b), the self-organized neuronal network has less ability to transfer neural information in local circuits, which results in a temporary decrease in local efficiency (Fig. 7c), similar to the results in Fig. 6c. However, as the coupling strength further increases, the number of weak synapses decreases but the number of strong synapses increases, accompanied by a increases in local efficiency, indicating the reconfiguration of synaptic connections. Compared to the network efficiency without magnetic fields (i.e., *k*_1_ = 0.0), the magnetic coupling always produces a higher global efficiency (Fig. 7d) and can also induce a higher local efficiency at suitable coupling strengths. These results reveal that spontaneous magnetic coupling between neurons promotes the formation of an economical neuronal network structure by reconfiguring the synaptic connections.

## Discussion

In this paper, we investigate how the brain neuronal network self-organizes into an economical structure under modulation by spontaneous electromagnetic induction. We first show that negative feedback from spontaneous magnetic fields slows down the self-organization process. Then, for suitable negative feedbacks, magnetic fields induce weaker firing synchronization by homogenizing the directed synaptic connections, where the connectivity patterns of self-organized neuronal networks have lower modularity, higher local and global efficiency. Finally, with suitable magnetic couplings, spontaneous magnetic fields can further homogenize the synaptic connections, decrease the modularity and enhance the local and glocal efficiency. Our results provide a comprehensive understanding of the significant effects of spontaneous electromagnetic induction on the self-organization process and the resulting modulation of the formation of an economical neuronal network.

The macroscopic dynamical behaviors in the neuronal network promote the formation of the self-organized structure due to the synaptic plasticity, which in turn produces different network dynamics. Thus, the topological properties of self-organized neuronal networks are closely related to the global dynamics, i.e. network synchronization. The modulation of chemical synapses by STDP is controlled by the firing time lag between the pre- and postsynaptic neurons^14^, i.e., the synchronization between neurons (see Eq. 7), and thus, the self-organization process will be faster with more rapid global synchronization. The negative feedback of the magnetic field inhibits the neuronal excitability so as to decrease the network synchronization^47,68^, it is thus reasonable that spontaneous electromagnetic induction would slow down the self-organization process. Meanwhile, the magnetic coupling between neurons can contribute to the signal exchange between neurons and promote the global synchronization of the neuronal network with a constant structure^45,47,51,52,66,69,70^, and thus could be another effective way for signal propagation in the network. But the increase in the magnetic coupling also produces higher negative feedback on neurons to inhibit the synchronization, ultimately inhibiting the self-organization process. Therefore, spontaneous electromagnetic induction slows down the self-organization process with weaker neuronal synchronization.

As a result of the slowly self-organization process under spontaneous electromagnetic induction, the neuronal network can contradictorily achieve high efficiency of information transmission on both the local and global scales from the perspective of graph theory. Indeed, the complex collective dynamics are formed from the connections between neurons and the topological structure of the neuronal network is crucial to effective communication of neural information^16,71,72^. Spontaneous electromagnetic induction weakens the update of synapses by decreasing the neuronal excitation, which would create more paths for neural information transmission in self-organized neuronal networks and produce high local and global efficiency. This modulation also generates higher loop numbers among neurons, supporting weaker global synchronization^73^. Note that the economical self-organized network provides a potentially structural ability to support the efficient information communication with low energy cost, and the structural economy is not the same to the economical cost of biophysical or biological energy consumed by neurons and synapses, but they have complex relationships^36,74–80^. Our results show an economical self-organized neuronal network structure induced by spontaneous electromagnetic induction, which intrinsically supports the weaker neuronal synchronization to transfer less information and may provide a structural foundation for the economic energy consumption of neurons and synapses. Finally, it needs to note that the precise timing of pre- and postsynaptic spikes determines the outcome of STDP rule, and the propagation delay of neural signals crucially affects the connectivity patterns and dynamics of self-organized neuronal networks^21,22^. Thus, the propagation delay would affect the results presented in this paper, which needs to be further investigated.

## Models and Methods

### Neuronal network model

We used the FitzHugh-Nagumo model to construct the neuronal network^18,81,82^:

1 $$\begin{array}{rcl}\varepsilon \frac{d{V}_{i}}{dt} & = & {V}_{i}-\frac{{{V}_{i}}^{3}}{3}-{W}_{i}+{I}_{ext}+{{I}_{i}}^{syn}+{{I}_{i}}^{mag}\\ \frac{d{W}_{i}}{dt} & = & {V}_{i}+a-{b}_{i}{W}_{i}\end{array}$$

where *V*_*i*_ is the membrane potential of the *i* th neuron and *W*_*i*_ is the corresponding membrane recovery variable. *ε*, *a* and *b*_*i*_ are dimensionless parameters, and *I*_*ext*_ is the external applied current. ${{I}_{i}}^{syn}$ is the total chemical synaptic current received by the *i* th neuron and is described as:

2 $$\begin{array}{rcl}{{I}_{i}}^{syn} & = & -\,\sum _{j(j\ne i)}^{N}\,{g}_{ij}{s}_{j}({V}_{i}-{V}_{syn})\\ \frac{d{s}_{j}}{dt} & = & \alpha ({V}_{j})(1-{s}_{j})-\beta {s}_{j}\\ \alpha ({V}_{j}) & = & {\alpha }_{0}/(1+{e}^{-{V}_{j}/{V}_{shp}})\end{array}$$

Here, *g*_*ij*_ is a measure of the synaptic weight from neuron *j* to neuron *i* and is modulated by the neuronal electric activities through the STDP rule. The synaptic variable *s*_*j*_ is controlled by the synaptic recovery and decay processes, i.e., *α*(*V*_*j*_)(1 − *s*_*j*_) and −*βs*_*j*_, where *α*(*V*_*j*_) is the synaptic recovery function and *β* is the decay rate. While the presynaptic membrane potential is below the threshold (i.e., *V*_*j*_ < 0 mV), the chemical synapse cannot be activated and subsequently decays with a rate of *β*, and thus, *s*_*j*_ can be reduced to *ds*_*j*_/*dt* = −*βs*_*j*_. Otherwise, the chemical synapse is strongly activated to act on the postsynaptic neuron, and *s*_*j*_ quickly jumps to 1. In addition, *V*_*syn*_ is the reversal potential of the chemical synapse and is related to the neurotransmitters and receptors; its value is set to 0 mV for excitatory synapses and to 2 mV for inhibitory synapses^16,18^.

### Model of spontaneous electromagnetic induction

The transmembrane transport of ions in neurons inevitably induces a temporally variable electromagnetic field^75,83^, which conversely exerts negative feedback on the neuronal electric activities^34,45^. A memristor model can be used to describe the physical correspondence between magnetic flux and electric charge and has recently been introduced to describe the coupling between a magnetic field and the membrane potential of a neuron^34,43,69^. The feedback current ${{I}_{i}}^{mag}$ induced by the magnetic field on the membrane potential is described as^34,69^:

3 $$\begin{array}{l}{{I}_{i}}^{mag}=-\,{k}_{1}\rho ({\varphi }_{i}){V}_{i}\end{array}$$

where the negative sign reflects the inhibitory effect of the magnetic field on the neuronal membrane potential and *k*_1_ represents the feedback strength. *ρ*(*ϕ*_*i*_) is the memory conductance of the memristor and is often chosen to be *ρ*(*ϕ*) = *c* + 3*dϕ*^2^, with constant parameters *c* and *d*. *ϕ*_*i*_ is a state variable of the magnetic field and satisfies:

4 $$\begin{array}{l}\frac{d{\varphi }_{i}}{dt}={k}_{3}{V}_{i}-{k}_{2}{\varphi }_{i}\end{array}$$

This equation illustrates that on the one hand, the magnetic field is driven by the electric activity of the neuron with driving strength *k*_3_, and on the other hand, it decays with a rate of *k*_2_ in the assumed homogeneous medium. In the neural system, spontaneous electric-magnetic fields are interacted among neurons, which provide a hidden spatial channel for the communication of neural information^45,47,52^. Considering the magnetic coupling between neurons, Eq. 4 can be rewritten as:

5 $$\begin{array}{l}\frac{d{\varphi }_{i}}{dt}={k}_{3}{V}_{i}-{k}_{2}{\varphi }_{i}+D\sum _{j}^{N}\,f({\varphi }_{j},{\varphi }_{i})\end{array}$$

Here, *D* represents the strength of the magnetic field coupling between neurons and *f*(*ϕ*_*j*_, *ϕ*_*i*_) describes the form of the coupling^70^. The magnetic coupling depends on many factors, such as the density of the medium and the spatial distance between neurons^45,47^. Here, we focus on the collective dynamics in a local neuronal circuit and thus assume a simple linear diffusive magnetic field coupling between neurons, i.e., *f*(*ϕ*_*j*_, *ϕ*_*i*_) = *ϕ*_*j*_ − *ϕ*_*i*_^47^.

### Spike-timing-dependent plasticity

When the presynaptic neuron *i* fires at time *t*_*i*_ and the postsynaptic neuron *j* fires at *t*_*j*_, the updating of the synaptic weight through the STDP rule is described as^16,17^:

6 $$\begin{array}{l}{\rm{\Delta }}{g}_{ij}={g}_{ij}F({\rm{\Delta }}t)\end{array}$$

with the updating function:

7 $$F({\rm{\Delta }}t)=\{\begin{array}{ll}{A}_{+}exp(-{\rm{\Delta }}t/{\tau }_{+}) & {\rm{if}}\,{\rm{\Delta }}t > 0\\ -{A}_{-}exp({\rm{\Delta }}t/{\tau }_{-}) & {\rm{if}}\,{\rm{\Delta }}t < 0\\ 0 & {\rm{if}}\,{\rm{\Delta }}t=0\end{array}$$

where Δ*t* = *t*_*i*_ − *t*_*j*_ is the firing time lag, *τ*_+_ and *τ*_−_ are the temporal windows for synaptic refinement, and *A*_+_ and *A*_−_ determine the maximum magnitude of the synaptic update. The firing time lag Δ*t* is measured within the temporal windows, and the synaptic update is performed once the temporal windows have passed. In addition, the synaptic weights are restricted to the range [0, *g*_*max*_] to ensure the convergence of the STDP algorithm.

The values of the remaining parameters utilized in the above models are *ε* = 0.08, *I*_*ext*_ = 0.1, *a* = 0.7, *α*_0_ = 2, *β* = 1, *V*_*shp*_ = 0.05, *c* = 0.1, *d* = 0.02, *A*_+_ = 0.05, *A*_−_ = 0.0525, *τ*_+_ = *τ*_−_ = 2, and *g*_*max*_ = 0.1. The parameter *b* decides the degree of excitability of neurons^16^. A small value of *b* corresponds to high neuronal excitability; thus, these neurons are highly dominant in the self-organization process and have a high out-synaptic strength^16^. To promote the competition among neurons, we define *b* to be uniformly distributed in [0.25, 0.95] to introduce heterogeneity in the neuronal network. The variable parameters *k*_1_, *k*_2_ and *k*_3_ control the negative magnetic feedback and can induce significantly different and complex dynamic patterns in neurons, such as instabilities and multiple frequencies^68^. Larger *k*_1_ and *k*_3_ values or smaller *k*_2_ values, in principle, all result in stronger negative feedback on neurons. Without considering the complex modulation of neuronal dynamics induced by spontaneous electromagnetic induction, we set *k*_2_ = 1 and *k*_3_ = 1 and vary *k*_1_ to control the negative feedback strength.

### Graph theory measures

#### Causal flow

In a directed weighted network, the causal flow of a node measures its causal influence on another node^61^ and is defined as:

8 $$\begin{array}{l}C{F}_{i}={{k}_{i}}^{out}-{{k}_{i}}^{in}\end{array}$$

Here, ${{k}_{i}}^{out}={\sum }_{j\in N}\,{w}_{ij}$ is the out-degree of the node, and ${{k}_{i}}^{in}={\sum }_{j\in N}\,{w}_{ji}$ is the corresponding in-degree, where *N* is the set of all nodes and *w*_*ij*_ is the connection weight from node *i* to node *j*.

#### Modularity

The modularity is a measure of the extent to which a network is divided into modules, such that the nodes within a module have dense connections but nodes in different modules have sparse connections. In a directed weighted network, the modularity is described as^84^:

9 $$\begin{array}{l}Q=\frac{1}{l}\sum _{i,j\in N}\,[{w}_{ij}-\frac{{{k}_{i}}^{out}{{k}_{i}}^{in}}{l}]{\delta }_{{m}_{i},{m}_{j}}\end{array}$$

where $l={\sum }_{i,j\in N}\,{w}_{ij}$ is the sum of all connection weights in the network, ${\delta }_{{m}_{i},{m}_{j}}$ is the Kronecker delta function, and *m*_*i*_ is the module containing node *i*. If nodes *i* and *j* are in the same module, ${\delta }_{{m}_{i},{m}_{j}}=1$; otherwise, ${\delta }_{{m}_{i},{m}_{j}}=0$.

#### Network efficiency

The local efficiency of a node is a measure of the information exchange among its neighbors; in a directed weighted network, this measure is defined as^84^:

10 $${E}_{local}=\frac{1}{2n}\sum _{i\in N}\,\frac{\sum _{j,h\in N,j,h\ne i}\,({w}_{ij}+{w}_{ji})({w}_{ih}+{w}_{hi})({[{d}_{jh}({N}_{i})]}^{-1}+{[{d}_{hj}({N}_{i})]}^{-1})}{({k}_{i}^{out}+{k}_{i}^{in})({k}_{i}^{out}+{k}_{i}^{in}-1)-2\sum _{j\in N}\,{w}_{ij}{w}_{ji}}$$

where *n* is the number of nodes and *d*_*jh*_(*N*_*i*_) is the shortest path from node *j* to node *h* that contains node *i*. The information exchange throughout the whole network is quantified by the global efficiency^84^:

11 $${E}_{glob}=\frac{1}{n}\sum _{i\in N}\,\frac{\sum _{j\in N,j\ne i}\,{d}_{ij}^{-1}}{n-1}$$

Here, all graph theory measures are calculated with the Brain Connectivity Toolbox (BCT, https://www.nitrc.org/projects/bct) in MATLAB, with no additional processing.

### Neuronal synchronization

The network synchronization is measured by a statistical factor of synchronization which is defined as follows^45,49^:

12 $$R=\frac{ < {F}^{2} > - < F{ > }^{2}}{\frac{1}{N}\sum _{i=1}^{N}\,( < {{V}_{i}}^{2} > - < {V}_{i}{ > }^{2})},$$

with

13 $$F=\frac{1}{N}\sum _{i=1}^{N}\,{V}_{i}.$$

Here, *N* is the number of neurons, *V*_*i*_ is the time series for neuron *i* in Eq. 1 and < >is the average over time. The *R* is in the range [0, 1]. *R* = 1 corresponds to the completely synchronous state, and the lower value reflects weaker network synchronization.
